# Aging in motion: how age and age simulation shape dual-task walking and memory

**DOI:** 10.1186/s11556-026-00426-w

**Published:** 2026-07-16

**Authors:** Anna Heggenberger, Patrick Harth, Sabine Schaefer, Christian Kaczmarek

**Affiliations:** Saarland University, Saarbrücken, Germany

**Keywords:** Age simulation suit, Dual-task performance, Cognitive-motor interference, Gait and mobility

## Abstract

**Background:**

Aging is accompanied by declines in cognitive, sensory, and motor functions that can adversely affect everyday activities, such as walking while performing a concurrent cognitive task. In this study, we investigate the effects of age and age simulation on dual-task performance, specifically focusing on gait and memory.

**Methods:**

We compare three groups: young adults (*n* = 28, *M*age = 21.82, *SD*age = 1.96), young adults wearing an age simulation suit (*n* = 28, *M*age = 22.07, *SD*age = 1.51), and older adults (*n* = 28, *M*age = 72.82, *SD*age = 6.74). Participants performed a 2-minute walking test (walking as fast as possible) and a word memory test (learning words presented on a piece of cardboard) under single- and dual- task conditions (repeated measures). Gait parameters, including walking speed, cadence, stride length, and double-support time were recorded using a wearable motion capture system. Mixed-design ANOVAs were conducted with Group as a between-subjects factor and Task Condition as a within-subjects factor.

**Results:**

The analyses revealed significant main effects of Task Condition, indicating impairments in both cognitive and gait performance under dual-task conditions (both *p* < .001), as well as significant main effects of Group, with older adults exhibiting the poorest performance overall (both *p* < .001). Notably, while memory performance did not differ significantly between young adults with and without the age simulation suit (*p* = .729), the suit resulted in significantly slower gait speed (*p* < .001) and alterations in other gait parameters approximating older adults’ gait profiles.

**Conclusions:**

These results underscore that declines in central attentional capacity combined with peripheral deficits contribute to dual-task interference, potentially increasing instability and fall risk in older populations. Importantly, the group differences reveal how these mechanisms contribute in different ways to age-related performance declines.

**Supplementary Information:**

The online version contains supplementary material available at 10.1186/s11556-026-00426-w.

## Background

Imagine older adults walking through a busy train station while reading departure times on an overhead display. As they scan the board, their pace slows, and their steps become more irregular. Performing two tasks at once can be challenging, potentially causing dual-task interference. The ability to efficiently coordinate cognitive and motor tasks deteriorates with age [1, 2], increasing the risk of falls [3].

Cognitive-motor dual-task paradigms typically involve the combination of a motor task, such as walking, with a cognitive task, such as word recall or verbal fluency [4–6]. Research consistently shows that older adults experience reduced performance in both task domains and greater dual-task impairments than younger adults [2, 7, 8]. For example, in a study by Lindenberger et al. [6], participants were asked to memorize word lists while walking on narrow tracks. Older adults performed worse than younger adults in both the memory and walking single-tasks. Moreover, these age-related differences became more pronounced under dual-task conditions, with older adults exhibiting a marked decrease in walking speed, increased step errors, and lower recall performance. Similar findings were reported by Brustio et al. [4], who observed greater declines with increasing adult age in gait speed and cognitive performance when completing verbal fluency and counting tasks while walking. To better understand the mechanisms underlying these impairments, it is essential to examine both cognitive and sensorimotor factors that contribute to poorer performance and dual-task interference in aging.

### Cognitive decline in aging

As individuals age, cognitive functions such as working memory, cognitive flexibility, and inhibitory control decline due to structural brain changes, including grey matter atrophy, cortical thinning, and white matter degradation [9, 10]. These alterations affect key regions involved in executive functions, such as the prefrontal cortex and hippocampus, leading to overall cognitive decline [8, 11]. Central processing capacity, which is critical for managing multiple concurrent tasks, is also affected by aging, resulting in less efficient allocation of attentional resources [12]. According to the Central Capacity Sharing Model, cognitive resources are finite and must be distributed across concurrent tasks [13]. With aging, not only does the overall resource pool diminish, but the efficiency of resource allocation also declines, further exacerbating the difficulties in multitasking [9, 14, 15]. Compensatory strategies, such as prioritizing gait stability to minimize fall risk, may be adaptive in old adulthood [16–18].

### Sensorimotor impairments in aging

Beyond cognitive limitations, aging is associated with significant sensorimotor changes. Visual and auditory declines reduce the ability to accurately perceive environmental cues, making it more challenging to maintain stability during dynamic tasks, particularly when attentional resources are divided [19, 20]. Moreover, sensorimotor impairments like reduced muscle strength in the lower limbs lead to slower walking speed, shorter stride length, and prolonged double-support time, as older adults adopt a more cautious gait to maintain stability [21, 22]. Additionally, declines in joint mobility limit the range of motion, making movements more effortful and less fluid [23, 24]. Reductions in flexibility restrict older adults’ ability to adjust gait patterns when attentional resources are diverted [25], and declines in proprioception render automatic postural adjustments less effective [26]. Under dual-task conditions, the diminished muscular reserve may restrict adaptive responses, increasing the risk of imbalance.

### Age simulation

Age simulation suits do not reproduce the complex and multifactorial nature of biological aging. Rather, they provide an experimental approach to impose peripheral constraints commonly associated with aging while preserving central cognitive processes, as they do not alter the neural integrity of the brain [27–29]. These suits mimic age-related sensorimotor constraints through weighted elements, joint mobility restrictors, and sensory impairments (e.g., blurred vision, reduced auditory input), mimicking declines in muscle strength, flexibility, and proprioception. Several studies have investigated the effects of age simulation suits on both motor and cognitive performance. For example, Vieweg and Schaefer [29] found that the age simulation suit reduced younger adults’ gross and fine motor performance to older adults’ levels. However, cognitive performance was not affected by the suit [30].

Few studies have examined the effects of age simulation suits specifically on gait. The age suit led to slower walking speed, shorter stride length, and increased step width [31–33]. However, these studies have focused on single-task walking, without additional cognitive demands. Laurentius et al. [32] asked young participants to walk on a treadmill with and without the suit, while older adults walked without it. Wearing the suit significantly slowed walking speed, reduced stride length, and increased stride time, partially mimicking the gait characteristics of older adults. While these findings highlight how simulated aging affects basic locomotion and cognition, little is known about its impact under dual-task conditions. Understanding whether simulated peripheral aging affects dual-task performance is of both theoretical and practical relevance. Age-related reductions in dual-task performance are often attributed to limited attentional resources and executive decline. However, peripheral changes such as reduced mobility, sensory degradation, and altered proprioception may also contribute to performance decrements, particularly when walking must be coordinated with a concurrent cognitive task. Distinguishing between these mechanisms is important because they may require different intervention approaches. While cognitive limitations may benefit from cognitive training, peripheral impairments may be addressed through physical exercise or mobility interventions. Age simulation suits provide an opportunity to experimentally isolate selected peripheral constraints while preserving central cognitive functioning and may therefore improve our understanding of the mechanisms underlying age-related dual-task difficulties.

The current study aims to disentangle the contributions of peripheral versus central aging to dual-task performance in gait and memory, by systematically comparing young adults, young adults wearing an age simulation suit, and older adults. We hypothesize main effects of group, with older adults showing the poorest performance, followed by young adults wearing the age simulation suit, and young adults without the suit performing best. As the suit simulates peripheral but not central limitations, we expect the suit to primarily affect gait rather than memory. We further predict a general dual-task effect, with performance declining from single- to dual-task across all groups. Moreover, we expect an interaction of group and performance declines, assuming that older adults show greater dual-task costs due to combined cognitive and sensorimotor decline.

## Method

### Power analysis

To estimate the required sample size for our study, we conducted an a priori power analysis using G*Power 3.1 for a mixed-design ANOVA with a within-between interaction. Assuming a medium effect size of *f* = 0.25, an alpha level of 0.05, a power of 0.95, and a correlation among repeated measures of *r* = .50, the analysis indicated that a total sample size of 66 participants would be required. Our final sample consisted of 84 participants, suggesting sufficient power to detect the expected interaction effect.

### Participants

We tested 28 young adults without the age simulation suit (*M*_*age*_ = 21.82, *SD* = 1.96, range = 18–28 years; 17 men and 11 women), 28 young adults wearing the suit (*M*_*age*_ = 22.07, *SD* = 1.51, range = 19–25 years; 11 men and 17 women), and 28 older adults (*M*_*age*_ = 72.82, *SD* = 6.74, range = 60–85 years; 14 men and 14 women). Young participants were students of Sports Science at [blinded for review], while older participants were recruited from local sports groups. The sample consisted of individuals of Caucasian European descent who lived in medium-sized towns across [blinded for review]. Exclusion criteria included neurological disorders, severe physical impairments, and medications affecting central nervous system functioning. All participants had normal or corrected-to-normal vision. Young adults participated for course credits, while older adults received monetary compensation (15 Euro).

### Tasks and materials

#### Cognitive task

The cognitive task asked participants to memorize a list of 20 words. Stimuli were 80 high-imagery, concrete, and age-fair nouns (e.g., Trommel [drum], Zebra [zebra], Pilot [pilot]), randomly assigned to four parallel word lists (20 words each). A different list was used in each trial, so that participants always memorized a new set of words. All words were presented in German on word cards (14 cm x 6.5 cm) in Calibri font (size 14). In each trial, participants were given 2 min to encode the 20 words. After this period, the word cards were returned, and participants walked to a desk and wrote down the memorized words.

#### Motor task

The motor task involved continuously walking as quickly as possible for 2 min along a straight path marked by two cones (length: 7 to 9 m). Gait speed and other gait parameters were recorded using the Mobility Lab™ system (APDM Wearable Technologies, Portland, USA). Six Opal inertial sensors were attached to participants at the sternum, lower back, wrists, and the dorsum of both feet [34]. Spatiotemporal gait parameters were automatically obtained from the Mobility Lab™ software, which processes recorded movement data using the manufacturer’s IWalk analysis algorithms. Walking speed was defined as the average forward walking velocity (m/s), cadence as the number of steps per minute, stride length as the distance between two consecutive foot contacts of the same foot (m), and double-support time as the percentage of the gait cycle during which both feet were simultaneously in contact with the ground. All parameters were directly exported from the software output, and no additional custom programming or processing of raw sensor data was performed.

#### Age simulation suit

**Fig. 1:**
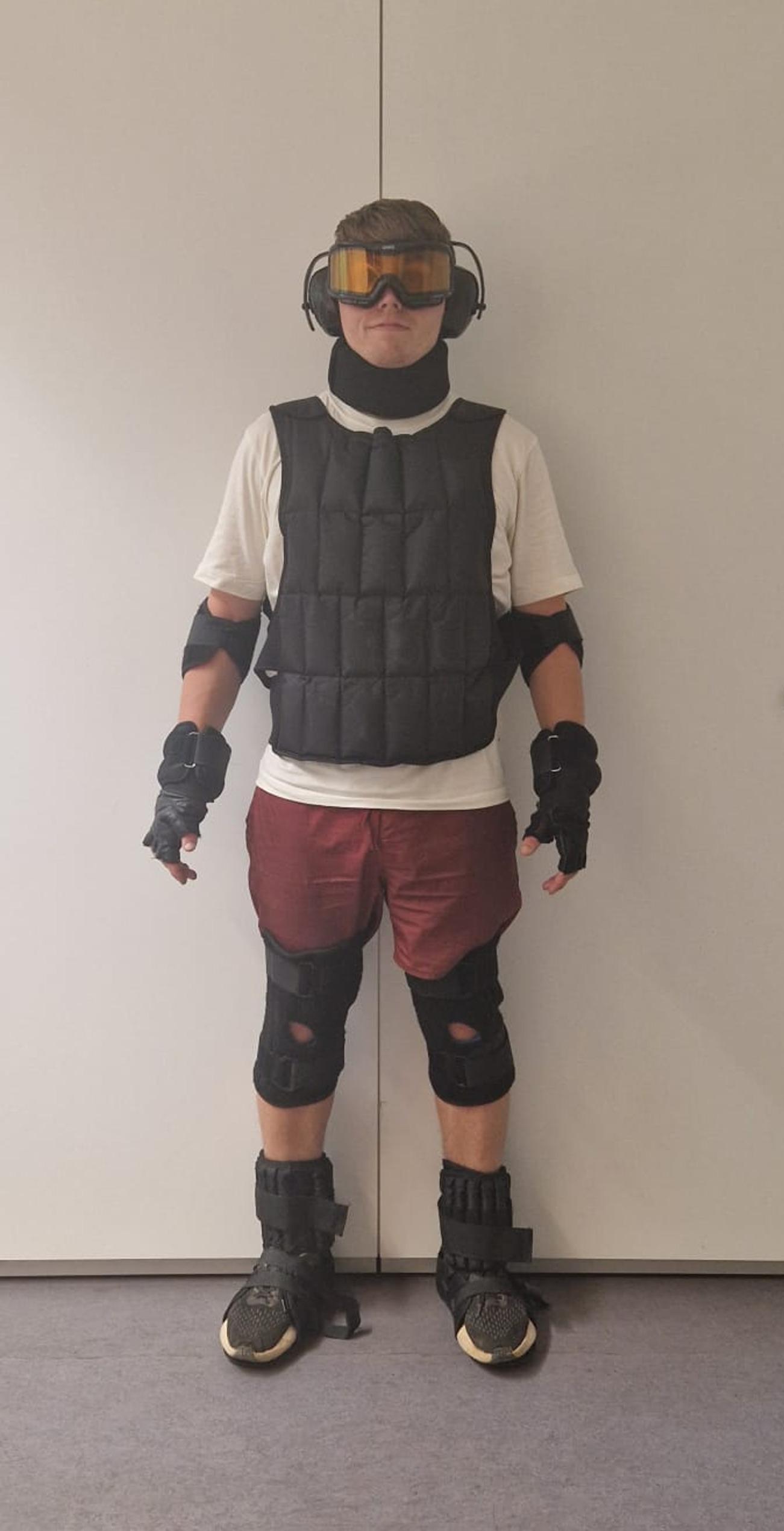
Age suit model “GERT” [28]

The GERT age simulation suit [28] was used to simulate peripheral sensory and motor impairments typically associated with aging (see Fig. 1). Special goggles induce blurred vision, alter colour perception, and narrow the visual field. Earmuffs mimic high-frequency hearing loss, while a neck brace restricts head and neck mobility. Knee and elbow bandages limit joint flexibility and range of motion, replicating the stiffness commonly observed in older adults. To simulate muscle weakness, the suit includes a weighted vest (10 kg) and additional limb weights (2.3 kg on each ankle, 1.5 kg on each wrist). Oversized overshoes with thick, soft foam soles reduce proprioceptive feedback and influence gait, and special gloves impair grip strength and fine motor control.

### Procedure

Upon arrival, participants completed questionnaires assessing demographic information, health status, sports engagement, and perceived physical state (see Supplement 1 for details). Young adults were randomly assigned to either the age simulation suit group or the control group without the suit. Participants in the suit condition put on the age suit before testing, and all participants were fitted with the APDM Mobility Lab sensors.

Participants completed the cognitive and motor tasks in a structured (single-dual-single) sequence. The structured sequence of single-task, dual-task, and single-task conditions was used to control for potential learning effects by obtaining measurements both before and after dual-task performance. Averaging the two single-task trials provided a more stable estimate of baseline performance. Participants first performed the memory task as a single-task, followed by the walking task as a single-task. In the following two dual-task trials, participants encoded the word list while walking for 2 min. They were instructed to walk as fast as possible while encoding as many words as possible. After that, participants completed one more single-task trial of the walking and the memory task.

The session concluded with a test of visual acuity, a cognitive screening, and a re-assessment of perceived physical state (see Supplement 1 and 2 for details), before removal of the Mobility Lab sensors and, if applicable, the age suit.

### Statistical analysis

The statistical analyses were conducted using R Statistical Software (Version 4.5.2) for Windows [35]. For each participant and outcome variable, the two single-task trials were averaged to obtain one single-task score, and the two dual-task trials were averaged to obtain one dual-task score. These aggregated scores were used for the mixed-design ANOVAs and for the calculation of dual-task costs.

Separate mixed-design ANOVAs were conducted for gait speed, memory performance, and each of the other gait parameters, with group (3: young adults, young adults wearing the age simulation suit, older adults) as a between-subjects factor and task condition (2: single-task, dual-task) as a within-subjects factor. Gait analyses were based on 26 participants in the group of young adults without the age simulation suit, due to technical difficulties during data acquisition.

Dual-task costs (DTCs) were calculated for both gait speed and memory performance using the averaged single-task and dual-task scores:

$$\:DTC=\left(\frac{Single\:task\:performance-Dual\:task\:performance}{Single\:task\:performance}\right)x\:100$$

To investigate group differences in dual-task costs, two separate mixed-design ANOVAs for cognitive and motor DTCs were conducted, with group (3) as the between-subjects factor.

For all analyses, post-hoc comparisons used Bonferroni correction to adjust for multiple comparisons. Statistical significance was set at α = 0.05. For all ANOVAs, assumptions of normality and homogeneity of variances were tested using Shapiro-Wilk and Levene’s tests. All assumptions were met, except for the ANOVA on DTCs in gait speed, where Levene’s test indicated unequal variances. Therefore, Welch’s ANOVA was used for this analysis, followed by Games-Howell post hoc tests for pairwise group comparisons.

## Results

### Memory performance

**Fig. 2:**
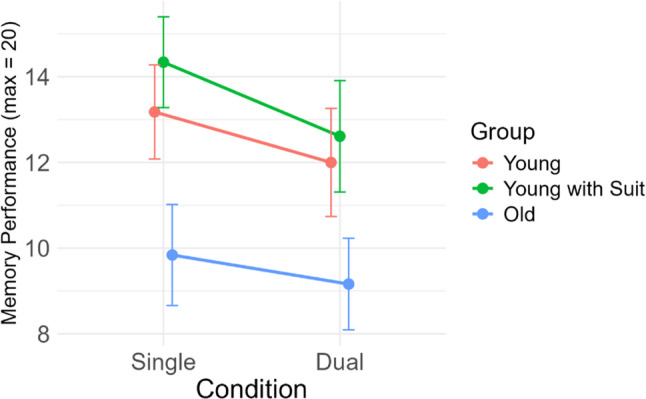
Memory performance across single- and dual-task conditions for each group Note: Single-task scores represent the mean of the two single-task trials conducted before and after the dual-task trials. Dual-task scores represent the mean of the two dual-task trials. Error bars depict 95% confidence intervals around the mean

Figure 2 illustrates memory performance across conditions (single-task vs. dual-task) for the three groups.

A mixed-design ANOVA was conducted to examine the effects of group and task condition on the number of correctly recalled words. The analysis revealed a significant main effect of group (*F*(2, 81) = 15.35, *p* < .001, *η*² = 0.27, 95% CI [0.14, 1.00]). A significant main effect of task condition was also observed (*F*(1, 81) = 27.66, *p* < .001, *η*² = 0.25, 95% CI [0.13,1.00]), indicating that memory performance was lower in the dual-task condition (*M* = 11.25, *SD* = 3.44) compared to the single-task condition (*M* = 12.45, *SD* = 3.42). The interaction effect between group and task condition was not significant (*F*(2, 81) = 1.78, *p* = .175), suggesting that the dual-task effect on memory performance was comparable across groups.

Post-hoc comparisons for the main effect of group showed that young adults without the age simulation suit (*M* = 12.60, *SD* = 3.08) recalled significantly more words than older adults (*M* = 9.50, *SD* = 2.90, *t*(81) = 5.28, *p* < .001, *d* = 1.03, 95% CI [0.64, 1.51]). Similarly, young adults wearing the age simulation suit (*M* = 13.48, *SD* = 3.15) also recalled significantly more words than older adults (*t*(81) = 4.10, *p* < .001, *d* = 1.31, 95% CI [0.94, 1.76]). Memory performance did not differ significantly between young adults with and without the age simulation suit (*t*(81) = -1.18, *p* = .729).

### Gait speed

**Fig. 3:**
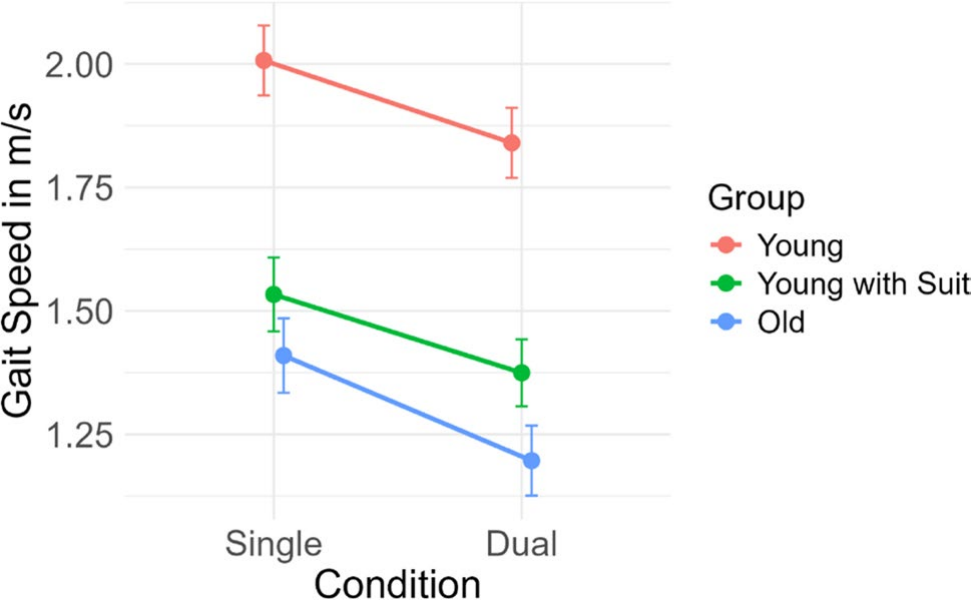
Gait Speed (m/s) across single- and dual-task conditions for each group. Note: Single-task scores represent the mean of the two single-task trials conducted before and after the dual-task trials. Dual-task scores represent the mean of the two dual-task trials. Error bars depict 95% confidence intervals around the mean

Figure 3 illustrates gait speed (m/s) across conditions (single-task vs. dual-task) for the three groups.

The mixed-design ANOVA for the effects of group and task condition on gait speed (m/s) revealed a significant main effect of group (*F*(2, 158) = 39.50, *p* < .001, *η*² = 0.20, 95% CI [0.11, 1.00]). In addition, there was a significant main effect of task condition (*F*(1, 158) = 166.69, *p* < .001, *η*² = 0.68, 95% CI [0.61, 1.00]), showing that gait speed was significantly lower in the dual-task condition (*M* = 1.46, *SD* = 0.32) compared to the single-task condition (*M* = 1.64, *SD* = 0.32, *p* < .001). The interaction between age group and task condition was not significant (*F*(2, 158) = 0.35, *p* = .703), indicating that the effect of dual-tasking on gait speed was similar across groups.

Post-hoc analyses for the main effect of group revealed that young adults without the age suit (*M* = 1.92, *SD* = 0.17) walked significantly faster than older adults (*M* = 1.30, *SD* = 0.18, *t*(158) = 17.59, *p* < .001, *d* = 3.03, 95% CI [2.54, 3.68]). Young adults wearing the age simulation suit (*M* = 1.45, *SD* = 0.18) also walked significantly faster than older adults (*t*(158) = 4.36, *p* < .001, *d* = 0.73, 95% CI [0.37, 1.11]). Furthermore, young adults without the age simulation suit walked significantly faster than those wearing the suit (*t*(158) = 13.31, *p* < .001, *d* = 2.39, 95% CI [1.96, 2.95]).

### Gait analyses

Table 1 presents additional parameters of the qualitative gait analysis. Note that young adults in the suit still outperform older adults in all gait parameters except cadence, where they show lower values than the other two groups.

**Table 1:** Descriptive statistics and ANOVA results for gait parameters across groups and conditions

	Single			Dual			Test Statistics
	Young (*n* = 26)	Young+Suit (*n* = 28)	Old (*n* = 28)	Young (*n* = 26)	Young+Suit (*n* = 28)	Old (*n* = 28 )	
Gait Parameters *M* (*SD*)							
Gait Speed [meter/second]	2.01 (±0.18)	1.53 (±0.19)	1.41 (±0.19)	1.84 (±0.18)	1.37 (±0.17)	1.2 (±0.18)	*Group: F*(2, 158) = 166.70, ***p*** **< .001**
							*Condition*: *F*(1, 158) = 39.50, ***p*** **< .001**
							*Interaction: F*(2, 158) = 0.70 *p* = .703
Cadence [steps/minute]	144.93 (±13.50)	117.64 (±8.04)	127.07 (±7.70)	138.17 (±12.15)	113.66 (±8.10)	119.22 (±8.54)	*Group: F*(2, 158) = 97.92, ***p*** **< .001**
							*Condition*: *F*(1, 158) = 16.15, ***p*** **< .001**
							*Interaction: F*(2, 158) = 0.57 *p* = .565
Double Support [%]	12.23 (±1.31)	15.41 (±1.91)	17.63 (±1.96)	12.9 (±2.12)	17.01 (±2.49)	20.18 (±3.78)	*Group: F(2*,* 155)* = 90.82, ***p*** **< .001**
							*Condition*: *F(1*,* 155)* = 17.91, ***p*** **< .001**
							*Interaction: F(2*,*155) = 1.98 p = .142*
Stride Length [meter]	1.67 (±0.11)	1.59 (±0.12)	1.33 (±0.17)	1.6 (±0.11)	1.45 (±0.11)	1.2 (±0.16)	*Group: F(2*,* 158)* = 109.85, ***p*** **< .001**
							*Condition*: *F(1*,* 158)* = 25.63, ***p*** **< .001**
							*Interaction: F(2, 158) = 0.79 p = .455*

Single-task values represent the mean of the two single-task trials conducted before and after the dual-task trials. Dual-task values represent the mean of the two dual-task trials. ANOVA results indicate the main effects of group and condition for each gait parameter. All pairwise comparisons for group were significant

Bold values indicate statistically significant results

### Dual-task costs

The DTCs for gait and memory are presented in Table 2. A one-way Welch ANOVA was conducted to examine group differences in DTC for gait speed. The analysis revealed a significant main effect of group (*F*(2, 47.78) = 6.60, *p* = .003, ω² = 0.18, 95% CI [0.03, 1.00]). Post-hoc comparisons indicated that older adults exhibited significantly higher gait speed DTCs compared to young adults without the age simulation suit (*MD* = 6.69, *p* = .002, 95% CI [2.23, 11.15]) and young adults wearing the age simulation suit (*MD* = 4.67, *p* = .026, 95% CI [0.48, 8.87]). No significant differences in DTCs were observed between young adults with and without the age simulation suit (*MD* = 2.02, *p* = .178).

Another one-way ANOVA was conducted to examine group differences in memory DTCs. DTCs for memory performance were comparable across groups (*F*(2, 81) = 1.24, *p* = .296).

**Table 2:** DTCs for gait speed and memory performance across groups

Group	DTC Gait Speed (*M* ± *SD*)	DTC Memory Performance (*M* ± *SD*)
Young without Suit (*n* = 28)	8.29% (± 4.67)	8.11% (± 20.91)
Young with Suit (*n* = 28)	10.31% (± 3.36)	12.44% (± 12.35)
Old (*n* = 28)	14.98% (± 8.42)	5.34% (± 16.69)

## Discussion

This study investigated how an age simulation suit affects walking and memory performance under single and dual-task conditions in young adults, and it compares their performances to those of older adults. In previous studies, age simulation suits have led to declines in gross motor performance and gait in single-task situations [29, 32]. As expected, older adults showed significantly lower performance than younger adults across both domains (memory and walking). Young adults wearing the age simulation suit demonstrated clear gait impairments, e.g., reduced walking speed and stride length, but their memory performance remained unaffected. Across all groups, memory and walking performance declined from single- to dual-task, reflecting a general dual-task effect. However, the magnitude of decline did not differ significantly between groups for either domain. Notably, older adults exhibited higher relative dual-task costs in gait compared to both younger groups, whereas no group differences emerged for dual-task costs in memory.

### Memory performance

Memory performance was generally lower in older adults compared to both groups of younger adults, regardless of task condition. Reduced processing speed, diminished working memory capacity, and decreased inhibitory control contribute to cognitive impairments in older adults, both in single- and dual-task scenarios [1, 8]. Notably, the age simulation suit did not affect memory performance. This indicates that the suit predominantly targets peripheral sensory and motor functions, such as mobility, joint flexibility, or visual and auditory acuity, while leaving central cognitive mechanisms unaffected [27, 29], allowing younger adults to compensate for the constraints of the suit. Interestingly, supplementary analyses indicated that visual acuity in young adults wearing the age simulation suit was reduced beyond the level observed in the older adult group (see Supplement 2). Despite this pronounced visual impairment, memory performance remained unaffected. One possible explanation is that participants compensated for reduced visual input by relying on intact cognitive resources and adaptive encoding strategies, thereby maintaining performance in the memory task. This interpretation is further supported by the supplementary cognitive assessments reported in Supplement 2, which showed no indication that wearing the age simulation suit impaired global cognitive status or processing speed in young adults.

The decline in memory performance from single- to dual-task was similar across all groups. Since the suit does not reduce the neural integrity of the brain, it does not affect central processing capacity, allowing young adults to effectively allocate cognitive resources during dual-tasking. Note that some previous studies reported greater cognitive dual-task costs in older as compared to young adults [4–6]. In the current study, older adults may have strategically prioritized the cognitive task, at the expense of walking speed.

Their higher dual-task costs in gait compared to both younger groups may suggest that older adults used a posture-second strategy [36, 37]. Future research needs to further disentangle the conditions under which task prioritization processes shift towards posture-first principles [16–18, 38].

Together, these findings support the notion that peripheral sensorimotor constraints alone may not be sufficient to induce the broader dual-task-related cognitive impairments typically observed in older adulthood.

### Gait speed

Gait speed was significantly lower in older adults compared to both groups of young adults, consistent with previous research demonstrating reduced muscle strength, limited joint mobility, impaired balance control, and diminished proprioceptive function in old adulthood [23, 24, 26]. Additionally, young adults wearing the age simulation suit also walked significantly slower than their unsuited peers, indicating that the suit induced a combination of peripheral constraints, including increased joint stiffness, additional physical load, and reduced sensory input. However, these effects cannot be attributed solely to age-related mechanisms, as the additional physical load imposed by the suit may itself contribute directly to reductions in gait performance [29, 39]. The supplementary analyses further showed that the age simulation suit induced visual impairments exceeding those observed in the older adult group (Supplement 2). Reduced visual input may have contributed to the slower walking speed and altered gait patterns by increasing uncertainty about the surrounding environment and requiring more cautious locomotion. Although young adults with the suit still outperformed older adults, their gait speed was significantly impaired. This suggests that while the age suit mimicks aspects of physical aging, young adults may still compensate more efficiently for the constraints due to intact central nervous system functioning, higher neuromuscular reserves and more efficient motor control systems [11, 12]. We argue that the impact of the suit may depend on task complexity, with more pronounced effects in tasks requiring greater motor control or coordination. The relatively simple walking task of the current study did not reduce gait speed in the suited group to the level observed in older adults. Nonetheless, the observed reduction shows that peripheral restrictions alone are sufficient to impair motor function, even in otherwise healthy young individuals.

Besides this, across all groups, gait speed declined under dual-task conditions compared to single-task walking, likely due to increased demands on attentional resources needed to simultaneously manage walking and the cognitive task [1, 40]. However, the degree of decline from single-task to dual-task was comparable between groups. This supports the notion that dual-task interference in gait is not solely driven by peripheral factors, but also by central attentional demands [1, 40]. Importantly, this finding did not support our initial hypothesis that older adults would exhibit disproportionately larger performance declines under dual-task conditions due to combined cognitive and sensorimotor impairments. Instead, the absence of a significant interaction suggests that all groups experienced comparable relative reductions when transitioning from single- to dual-task conditions. Interestingly, older adults nevertheless showed higher motor dual-task costs than both younger groups. This pattern may reflect differences between absolute and relative measures of performance. While the transition from single- to dual-task conditions resulted in similar absolute performance reductions across groups, older adults started from a lower baseline gait speed. Consequently, comparable absolute changes translated into proportionally larger relative costs. This suggests that increased dual-task costs in older adults may partly reflect reduced baseline functional capacity rather than stronger dual-task interference per se.

### Gait analyses

Compared to the young adults without the suit, young adults wearing the suit showed slower gait speed, reduced cadence, shorter stride length, and increased double support time, both under single- and dual-task conditions. These changes mirror the typical gait alterations observed in aging populations [21, 22], but gait parameters of young adults in the suit were still significantly different from older adults. This pattern again suggests that while the simulation suit successfully mimics physical constraints of aging, younger individuals might benefit from intact central nervous system functioning and greater motor adaptability, enabling them to partially compensate for the imposed limitations [41]. Interestingly, cadence was the only parameter in which suited young adults performed worse than older adults. We assume that this was due to the added inertia caused by the ankle weights of the age simulation suit. With the current study design, specific effects of added physical load cannot be disentangled from other suit components, such as restricted joint mobility or altered sensory input. The increased effort required to initiate and complete each leg swing probably prolonged the swing phase, resulting in fewer steps per minute. Disentangling the influence of specific components of the suit on individual gait parameters requires further research.

In line with these motor effects, only the young adults wearing the suit also reported a marked decline in their perceived physical state across the session, whereas the other groups remained stable (detailed results are provided in Supplement 1). This subjective deterioration further supports the notion that the suit induces noticeable physical strain and bodily constraints, even though cognitive performance remained unaffected.

### Limitations

This study has limitations that should be acknowledged. One limitation concerns the ecological validity of the age simulation suit. Previous literature has highlighted that the ability of age simulation suits to reproduce the complex and systemic processes underlying biological aging remains limited [39]. The suit cannot reproduce long-term neurophysiological adaptations, sarcopenia, fatigue, or motivational changes. In addition, the suit combines multiple constraints, including restricted joint mobility, reduced sensory input, and substantial additional physical load. The present design does not allow disentangling the contribution of added weight from other suit components, such as restrictive bandages or altered visual and proprioceptive feedback. Although limb weights may contribute to slower walking speed, reduced cadence, and shorter stride length by increasing external load and inertial demands, they do not directly reflect gradual age-related declines in muscle function. Therefore, the suit should not be interpreted as a comprehensive simulation of aging. Instead, it should be regarded as an experimental manipulation of peripheral constraints associated with aging. Consequently, the present findings provide insight into how imposed sensory and motor constraints influence gait and dual-task performance, but they cannot fully explain the mechanisms underlying biological aging. Another limitation concerns the fixed order of task administration. Although the single-dual-single sequence was selected to account for potential learning effects by obtaining single-task measurements both before and after dual-task performance, a fixed order may still introduce practice or fatigue effects. To address this concern, supplementary analyses comparing the first and second single-task trials for memory performance and gait speed were conducted. These analyses did not indicate significant changes between the two single-task trials. Thus, there was no evidence for systematic practice or fatigue effects, suggesting that the fixed task order likely did not substantially influence the main findings (Supplement 3).

Participants were free to use different strategies to encode the word list, which have not been assessed systematically in the current study. Looking at the cardboard with the word list temporarily diverts attention away from the walking task, and participants may have done this less frequently under dual-task conditions. Future research should measure how the use of such strategies changes while dual-tasking, and whether the age suit influences these preferences as well.

Another limitation concerns the characteristics of the older adult sample. Older participants were recruited from local sports groups and additionally met several health-related inclusion criteria, including the absence of neurological disorders, severe physical impairments, and medications affecting central nervous system functioning. Consequently, the sample likely represented relatively healthy and physically active older adults with above-average functional capacities. Such a selection may have reduced age-related differences and dual-task impairments compared to those expected in the broader older adult population. Therefore, caution is warranted when generalizing the present findings to older individuals with lower physical activity levels, chronic conditions, or greater functional limitations.

## Conclusion

This study extended previous research by examining the effects of an age simulation suit on gait and memory under single-task and dual-task conditions. The suit induced gait impairments that closely mirrored age-related motor changes. These effects were present in single- and dual-task situations, indicating that peripheral constraints disrupt gait patterns. However, memory performance in suited young adults remained comparable to that of their unsuited peers, suggesting that intact central cognitive resources can compensate for simulated peripheral impairments.

All groups showed performance reductions in dual-task situations. Older adults exhibited significantly higher relative dual-task costs in gait compared to younger adults, possibly prioritizing cognitive performance at the expense of motor control. However, these effects should be interpreted in the context of lower baseline gait performance, as comparable absolute performance declines across groups did not support stronger dual-task interference in older adults. In contrast, suited young adults maintained gait performance more effectively, possibly due to preserved central processing and greater cognitive flexibility.

Taken together, the present findings suggest that experimentally imposed peripheral sensorimotor constraints are sufficient to substantially impair gait performance and induce gait characteristics approaching those of older adults, even in healthy young individuals. In contrast, cognitive performance remained largely unaffected by the age simulation suit, and the suit did not induce disproportionately stronger dual-task costs. This dissociation is theoretically important because it suggests that peripheral sensorimotor constraints alone cannot fully explain age-related dual-task difficulties. Rather, broader impairments in dual-task adaptation in older adulthood may additionally depend on central cognitive and attentional changes that are not reproduced by the suit.

## Supplementary Information

Supplementary Material 1.

Supplementary Material 2.

Supplementary Material 3.

## Data Availability

The dataset supporting the conclusions of this article is available in the Open Science Framework (OSF) repository, https://osf.io/zjxqb/?view_only=d9fc13cb3072439dac3bc635763c3162.This study was not preregistered. All materials necessary to evaluate and, where applicable, replicate the study procedures are available in the same OSF project. The de-identified primary dataset underlying all reported analyses is provided within the same OSF project, and the analysis scripts used to generate the reported results are also included.
