# Supplementary material for: Aging in motion: how age and age simulation shape dual-task walking and memory
Source: Eur Rev Aging Phys Act. 2026 Jul 16;23:29. doi: 10.1186/s11556-026-00426-w (PMC13386631; doi:10.1186/s11556-026-00426-w)
Supplement: Supplementary file 1 — Supplementary Material 1. [file 11556_2026_426_MOESM1_ESM.docx]

**Repeated Assessment of Perceived Physical State**

Descriptive statistics for age, height, and weekly sport activity in each group are presented in Table S1.1.

**Table S.1.1**
Demographic characteristics of the three groups (Mean ± SD)

|  | Young adults without suit | Young adults with suit | Old adults |
| --- | --- | --- | --- |
| Age | 21.82 (1.96) | 22.07 (1.51) | 72.82 (6.74) |
| Height in cm | 175.05 (9.41) | 177.00 (11.31) | 169.68 (7.18) |
| Sport minutes/week | 92.78 (36.04) | 100.45 (30.37) | 61.94 (33.12) |

The current physical state of the participants was assessed at the beginning and end of each testing session with the Perceived Physical State (“PEPS”) [1], a questionnaire of 20 adjectives representing four dimensions: physical energy (e.g., flabby, washed out), physical fitness (e.g., well trained, strong), physical flexibility (e.g., flexible, elastic), and physical health (e.g., sick, injured; [1]). The perceived physical state was assessed with a six-point rating scale ranging from not at all (0) to totally (5). The questionnaire was completed at the beginning and at the end of the session. Participants who wore the age simulation suit during testing also completed the post-assessment while still wearing the suit. To examine subjective physical state, repeated-measures ANOVAs were conducted separately for each of the four WKV subscales: Flexibility, Health, Fitness, and Energy. Each analysis included the within-subjects factor Time (2: pre vs. post) and the between-subjects factor Group (3: Young, Young with Suit, Old).

**Figure S1.1**

Perceived Energy Before and After Testing by Group


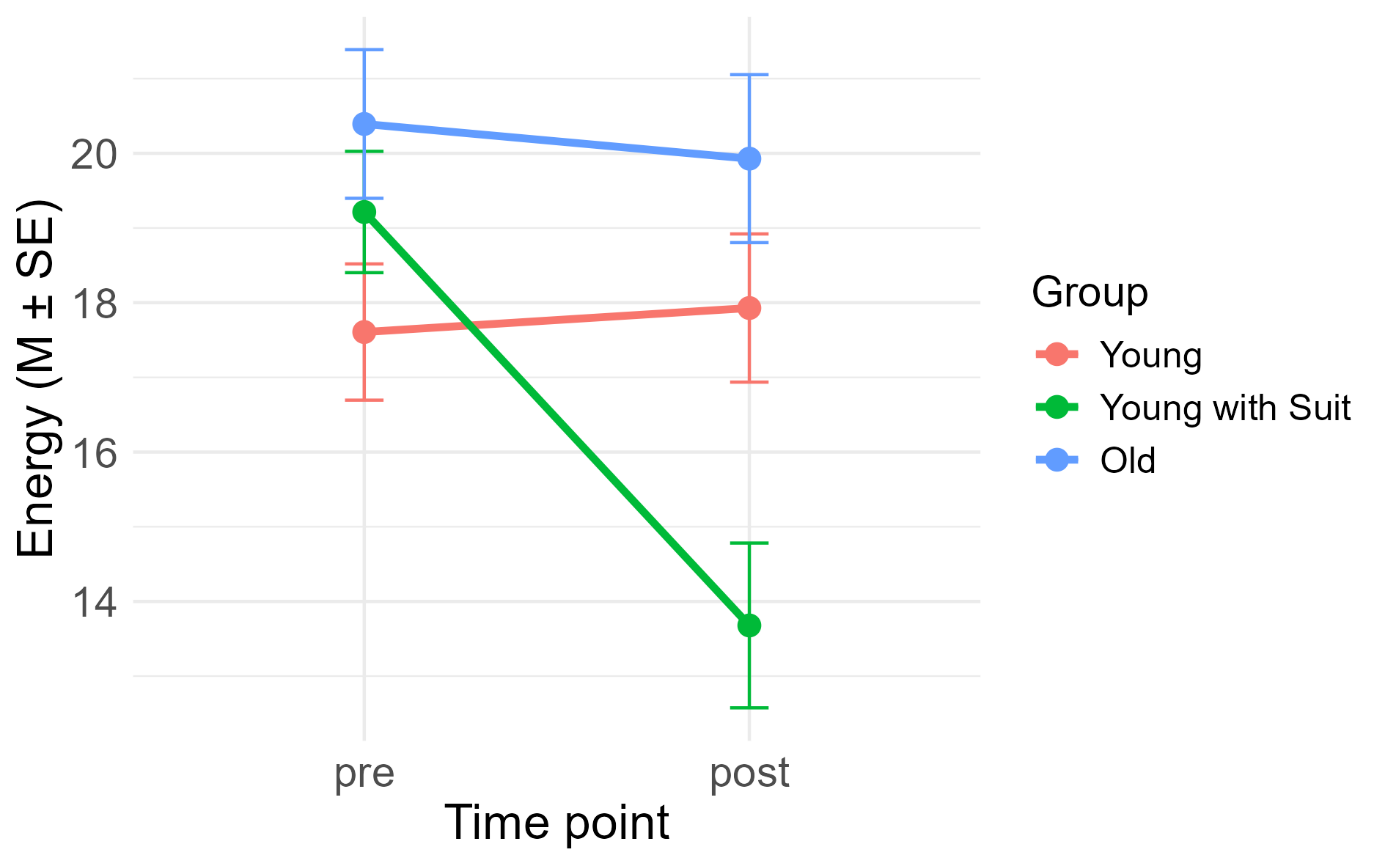


*Note.* Error bars depict SE mean.

For the subscale Energy, the analysis revealed a significant main effect of Group (*F*(2, 81) = 5.22, *p* = .007), a significant main effect of Time (*F*(1, 81) = 8.60, *p* = .004), and a significant Group × Time interaction (*F*(2, 81) = 8.09, *p* < .001). As shown in Figure S1.1, perceived energy decreased markedly from pre- to posttest in the Young with Suit group. This interaction suggests that wearing the age simulation suit led to a pronounced subjective decline in perceived energy.

**Figure S1.2**

*Perceived Fitness Before and After Testing by Group*


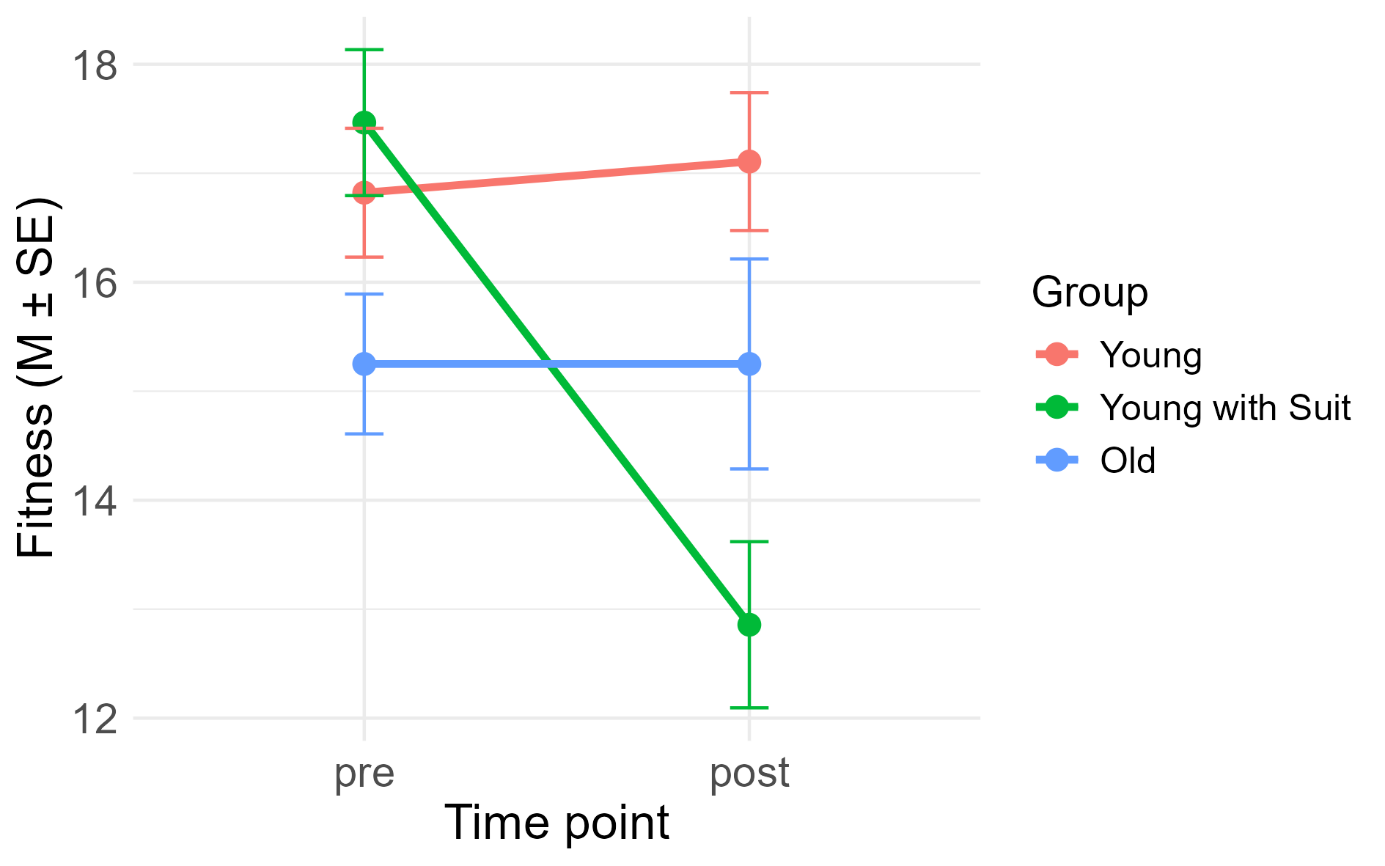


*Note.* Error bars depict SE mean.

For the subscale Fitness, the repeated-measures ANOVA revealed a significant main effect of Time (*F*(1, 81) = 12.29, *p* = .001), and a significant Group × Time interaction (*F*(2, 81) = 14.89, *p* < .001). The main effect of Group did not reach statistical significance (*F*(2, 81) = 2.63, *p* = .078). As shown in Figure S1.2, perceived fitness strongly declined from pre- to posttest in the Young with Suit group, while the Young and Old groups remained stable or showed slight increases. This pattern indicates that the suit had a substantial impact on participants’ subjective fitness perception.

**Figure S1.3**

*Perceived Health Before and After Testing by Group*


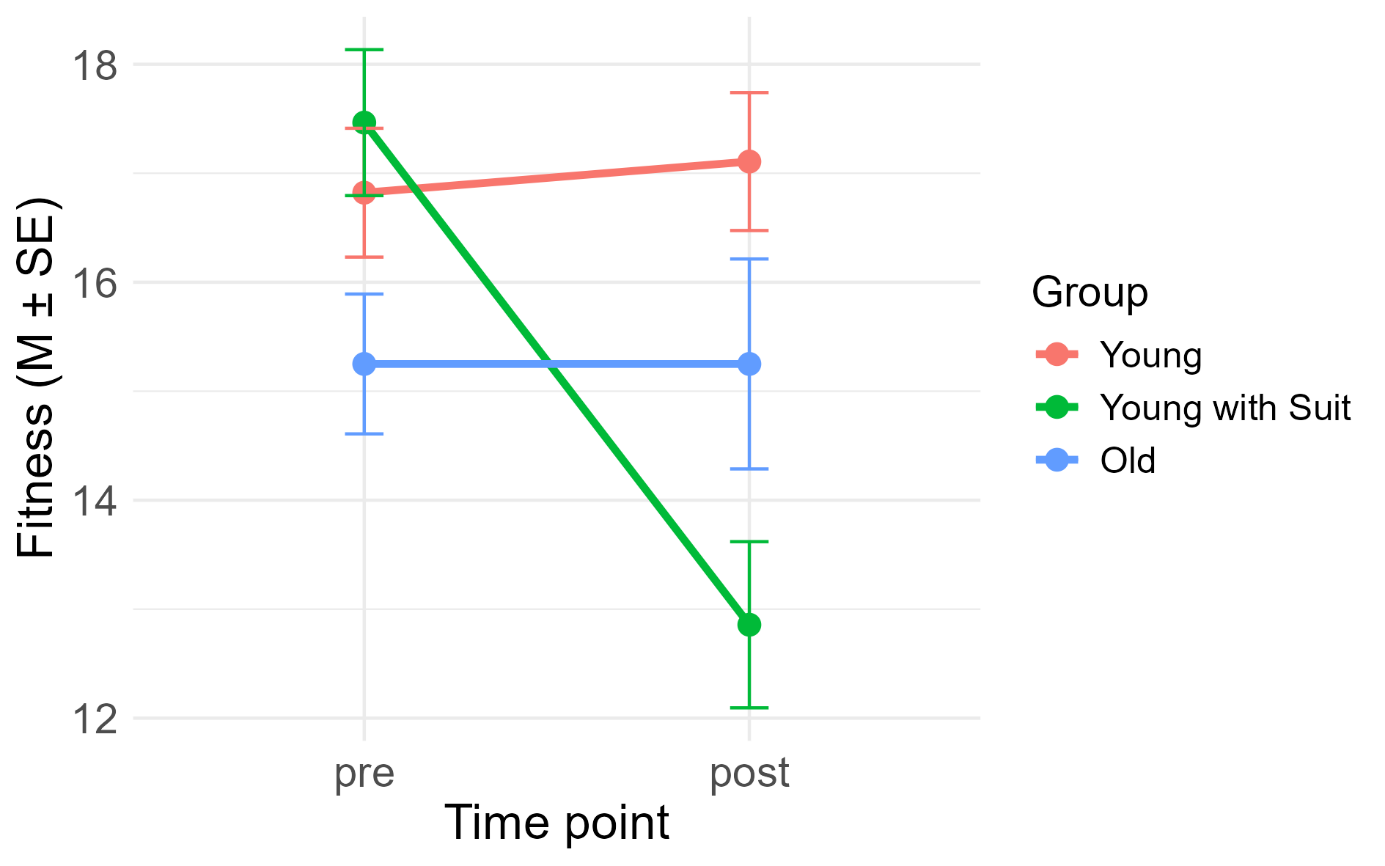


*Note.* Error bars depict SE mean.

For the subscale Health, the repeated-measures ANOVA showed no significant main effects of Group (*F*(2, 81) = 0.14, p = .869), or Time (*F*(1, 81) = 1.03, *p* = .312). However, the Group × Time interaction reached significance (*F*(2, 81) = 4.71, *p* = .012). As shown in Figure S1.3., the Young with Suit group reported a decrease in perceived health from pre- to posttest, whereas the Young and Old group showed an increase over time.

**Figure S1.4**

*Perceived Flexibility Before and After Testing by Group*


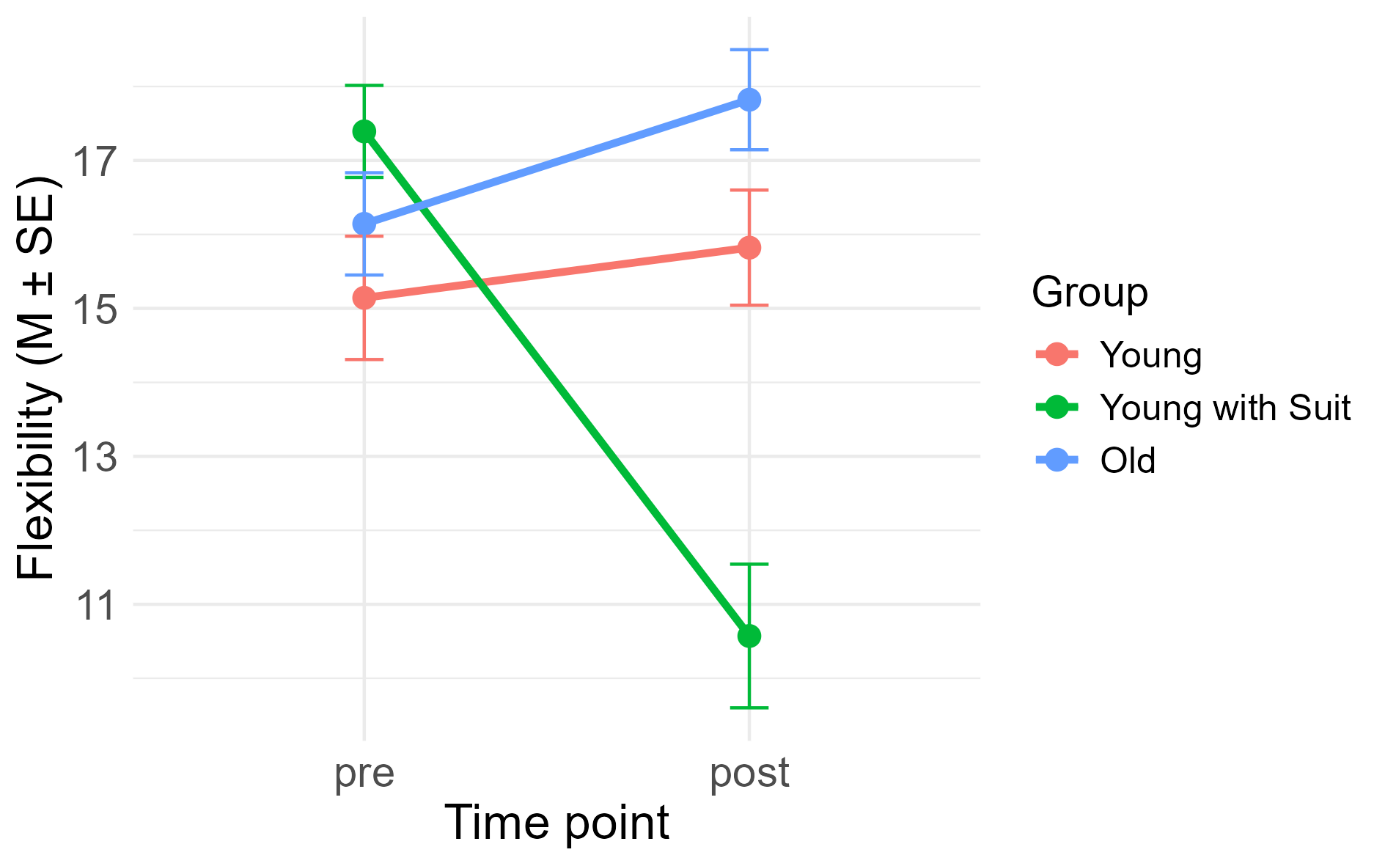


*Note.* Error bars depict SE mean.

For the subscale Flexibility, the repeated-measures ANOVA revealed a significant main effect of Group (*F*(2, 81) = 4.70, *p* = .012), a significant main effect of Time (*F*(1, 81) = 14.37, *p* < .001), and a significant Group × Time interaction (*F*(2, 81) = 46.69, *p* < .001). As depicted in Figure S1.4, perceived flexibility decreased substantially in the Young with Suit group, while both the Young and Old groups reported slight increases. This pattern indicates that wearing the age simulation suit led to a marked decline in perceived flexibility from pre- to posttest.

The findings from the WKV assessment demonstrate that wearing the age simulation suit led to a notable decline in perceived physical state among young adults. Specifically, participants in the Young with Suit group reported significantly lower levels of perceived energy, fitness, flexibility, and health after the testing session. Note that similar findings were obtained in an age simulation study by Schaefer et al. [2]. This pattern indicates that the acute, externally imposed sensorimotor constraints of the suit were subjectively experienced as physically exhausting and limiting. In contrast, the Old group showed stable or even slightly improved ratings from pre- to posttest. This may reflect either a habituation to their chronic physical limitations or a sense of accomplishment after successfully completing the demanding task. These differential patterns underscore that while the suit effectively simulates peripheral physical aging, its sudden and unfamiliar constraints likely produce stronger subjective strain in younger individuals who are not accustomed to such limitations, and wearing the suit may also trigger age stereotypes and negative projections of one’s own future aging (for a discussion, see also [2]).

**References**

1. Kleinert, J., 2006. Adjektivliste zur Erfassung der wahrgenommenen körperlichen Verfassung (WKV) [Adjective list for assessing perceived physical state]. Z. Sportpsychol. 13(4), 156–164. https://doi.org/10.1026/1612-5010.13.4.156

2. Schaefer, S., Bill, D., Hoor, M., & Vieweg, J., 2023. The influence of age and age simulation on task-difficulty choices in motor tasks. Aging Neuropsychol. Cogn. 30(3), 429–454. https://doi.org/10.1080/13825585.2022.2043232
