# Supplementary material for: Aging in motion: how age and age simulation shape dual-task walking and memory
Source: Eur Rev Aging Phys Act. 2026 Jul 16;23:29. doi: 10.1186/s11556-026-00426-w (PMC13386631; doi:10.1186/s11556-026-00426-w)
Supplement: Supplementary file 2 — Supplementary Material 2. [file 11556_2026_426_MOESM2_ESM.docx]

**Visual Acuity Testing and Cognitive Screening**

To assess visual and cognitive functioning, participants completed a near and far visual acuity test using standardized Landolt C charts [1]. For near vision, the chart was held at approximately 40 cm distance, for far vision, it was positioned at 3 meters. Following the visual tests, all participants underwent cognitive screening using the Montreal Cognitive Assessment (MoCA; [2]) and the Digit Symbol Substitution Test (DSST; [3]). The MoCA provided a brief evaluation of general cognitive functioning, while the DSST assessed processing speed and cognitive flexibility. Note that participants in the suit condition kept the suit on during visual acuity testing and cognitive screening.

**Results Visual Acuity Testing**

As illustrated in Figure S2.1 and Figure S2.2, clear group differences emerged in both near and far visual acuity.

**Figure S2.1**

Near Visual Acuity across Groups


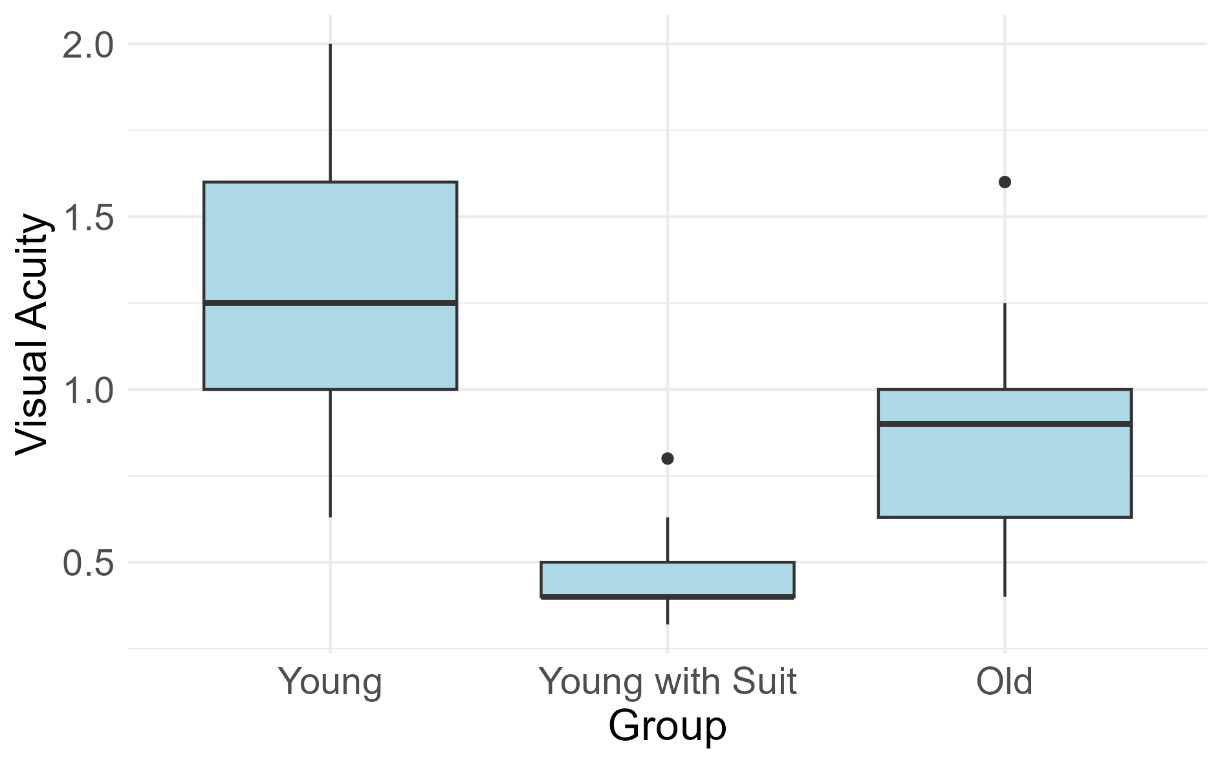


**Figure S2.2**

Far Visual Acuity across Groups


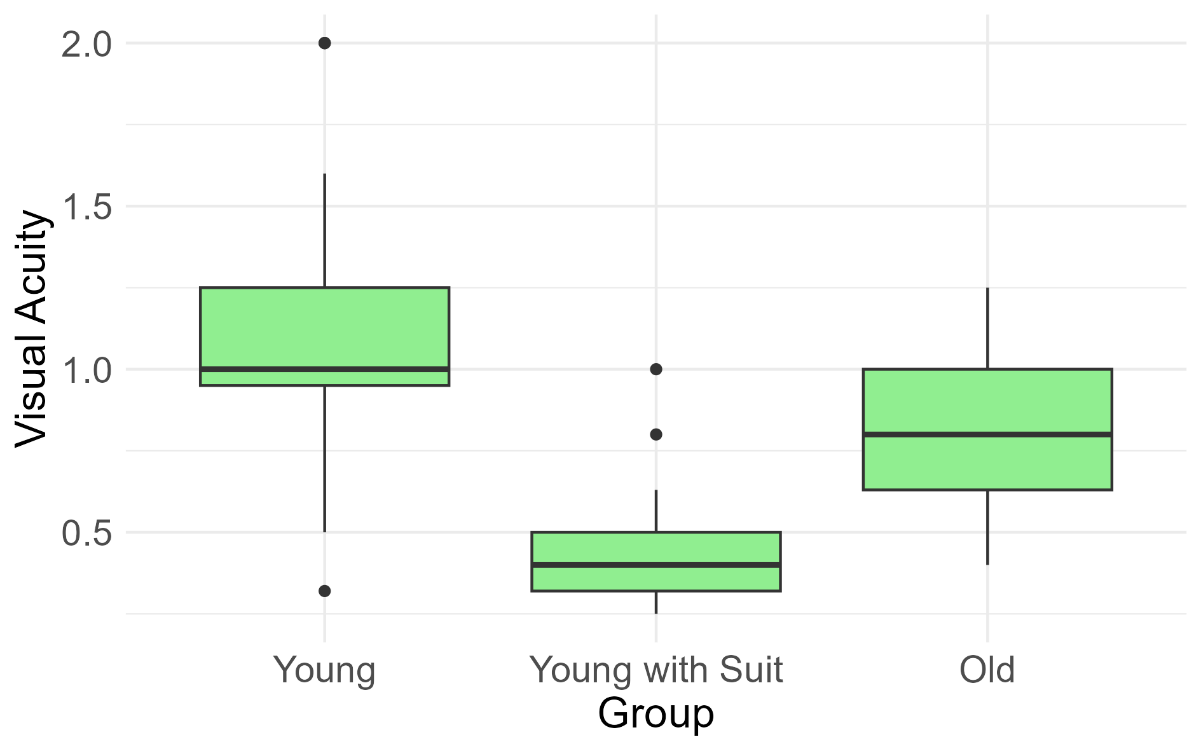


To examine group differences in visual acuity, two separate one-way ANOVAs were conducted for near and far visual acuity. For near visual acuity, the analysis revealed a significant main effect of group (*F*(2, 81) = 60.29, *p* < .001). Post-hoc comparisons (Tukey HSD) showed that the Young with Suit group had significantly lower visual acuity than both the Young (*p* < .001) and Old (*p* < .001) groups. The Old group also performed significantly worse than the Young group (*p* < .001), but better than the Young with Suit group.

Similarly, the ANOVA for far visual acuity yielded a significant main effect of group (*F*(2, 81) = 38.02, *p* < .001). Tukey post-hoc tests again indicated that the Young with Suit group performed significantly worse than both the Young (*p* < .001) and Old (*p* < .001) groups. The Old group also showed lower far visual acuity than the Young group (*p* < .001).

These findings confirm that wearing the age simulation suit significantly impairs visual acuity, simulating sensory declines commonly observed in older adults. Interestingly, the visual acuity of young participants wearing the suit was even lower than that of the older adults, highlighting the pronounced effect of the visual restriction components.

**Results Cognitive Screening**

Figures S2.3 and S2.4 illustrate the group differences in performance on the DSST and the MoCA.

**Figure S2.3**

Digit Symbol Score across Groups


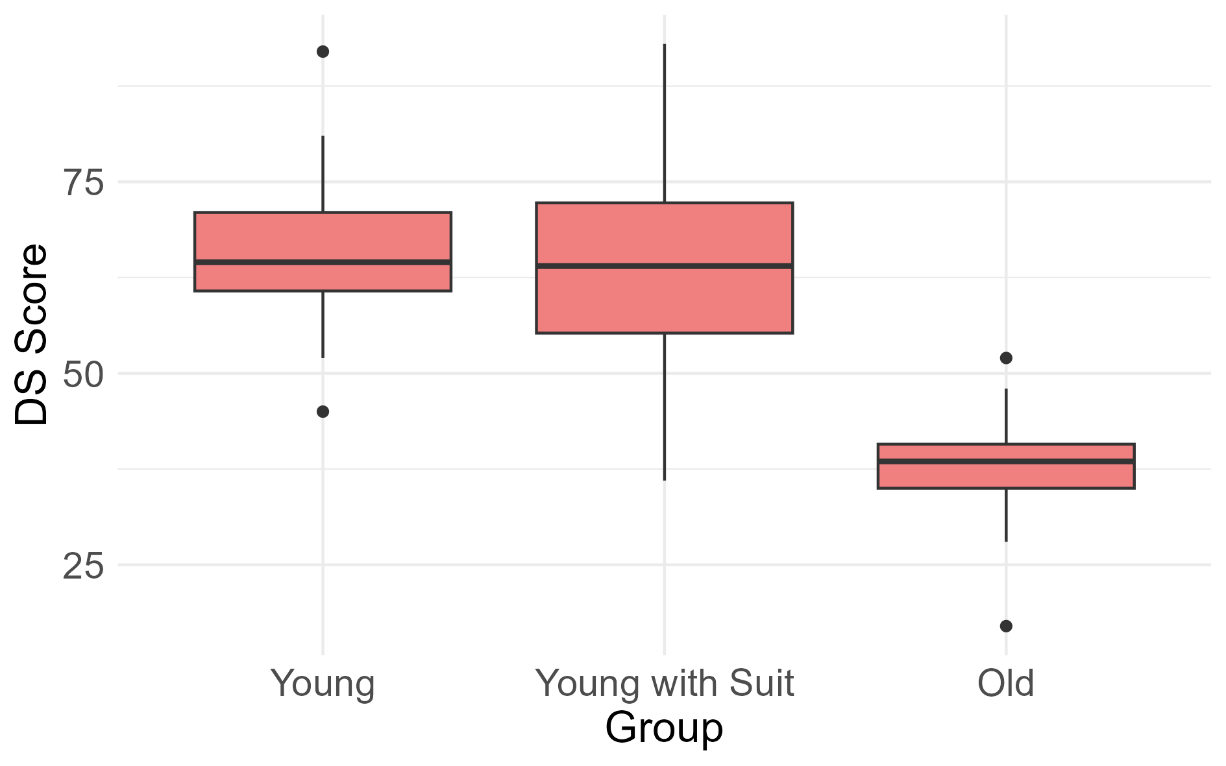


**Figure S2.4**

*MoCA Score across Groups*


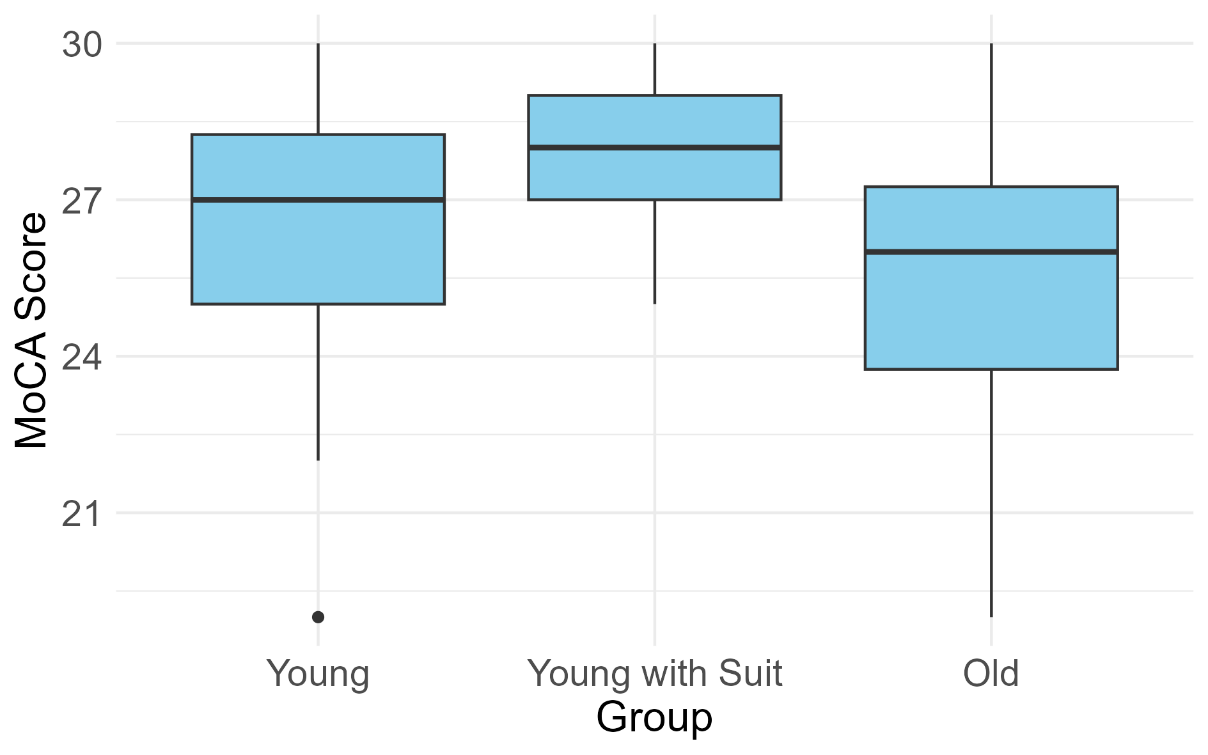


To statistically examine these differences, two separate one-way ANOVAs were conducted.

For the DSST, the analysis revealed a significant main effect of group (*F*(2, 81) = 69.26, *p* < .001). Post-hoc tests showed that the Old group performed significantly worse than both the Young (*p* < .001) and the Young with Suit group (*p* < .001), who did not differ significantly from each other (*p* = .93).

For the MoCA, the ANOVA also yielded a significant main effect of group (*F*(2, 81) = 7.34, *p* = .001). Post-hoc comparisons indicated that the Old group scored significantly lower than the Young with Suit group (*p* < .001), while neither comparison between the Young and Old nor between the Young and Young with Suit group reached significance (*p* > .05).

These findings suggest that the age simulation suit did not impair cognitive performance in young adults, likely because it primarily induces peripheral sensorimotor constraints without affecting central cognitive processing. The preserved cognitive resources in younger adults may have enabled them to compensate for the peripheral limitations imposed by the suit. This aligns with previous results showing that the suit impairs mainly motor performance, while cognitive functioning remains unaffected [4,5].

**References**

1. Precision Vision, 2021. Landholt C. https://www.precision-vision.com/product-category/etdrs/etdrs-charts/other-etdrs-charts/landolt-c-other-etdrs-charts/

2. Nasreddine, Z. S., Phillips, N. A., Bédirian, V., Charbonneau, S., Whitehead, V., Collin, I., Cummings, J. L., & Chertkow, H., 2005. The Montreal Cognitive Assessment, MoCA: A brief screening tool for mild cognitive impairment. J. Am. Geriatr. Soc. 53(4), 695–699. https://doi.org/10.1111/j.1532-5415.2005.53221.x

3. Wechsler, D., 1981. The psychometric tradition: Developing the Wechsler Adult Intelligence Scale. Contemp. Educ. Psychol. 6(2), 82–85. https://doi.org/10.1016/0361-476X(81)90035-7

4. Heggenberger, A., Vieweg, J., & Schaefer, S., in press. Age simulation effects on full-body motor sequence learning. Psychol. Aging.

5. Vieweg, J., Panzer, S., & Schaefer, S., 2023. Effects of age simulation and age on motor sequence learning: Interaction of age-related cognitive and motor decline. Hum. Mov. Sci. 87, 103025. https://doi.org/10.1016/j.humov.2022.103025
