# Supplementary material for: Aging in motion: how age and age simulation shape dual-task walking and memory
Source: Eur Rev Aging Phys Act. 2026 Jul 16;23:29. doi: 10.1186/s11556-026-00426-w (PMC13386631; doi:10.1186/s11556-026-00426-w)
Supplement: Supplementary file 3 — Supplementary Material 3. [file 11556_2026_426_MOESM3_ESM.docx]

**Evaluation of potential order effects**

To evaluate whether the fixed single-dual-single task sequence introduced systematic practice or fatigue effects, supplementary analyses were conducted comparing the first and second single-task trials for both memory performance and gait speed.

For memory performance, a paired-samples t-test revealed no significant difference between the first and second single-task trials (*t*(83) = −0.55, *p* = .581), indicating stable performance across measurements.

Similarly, gait speed did not differ significantly between the first and second single-task trials (*t*(81) = −0.19, *p* = .848). Figures S3.1 and S3.2 additionally illustrate the progression of memory performance and gait speed across all four task phases (Single 1, Dual 1, Dual 2, Single 2) separately for the three participant groups. Visual inspection supports the statistical findings and does not suggest systematic changes between the first and second single-task trials.

**Figure S3.1**

*Memory performance across the four task phases (Single 1, Dual 1, Dual 2, Single 2) for the three groups.*

*
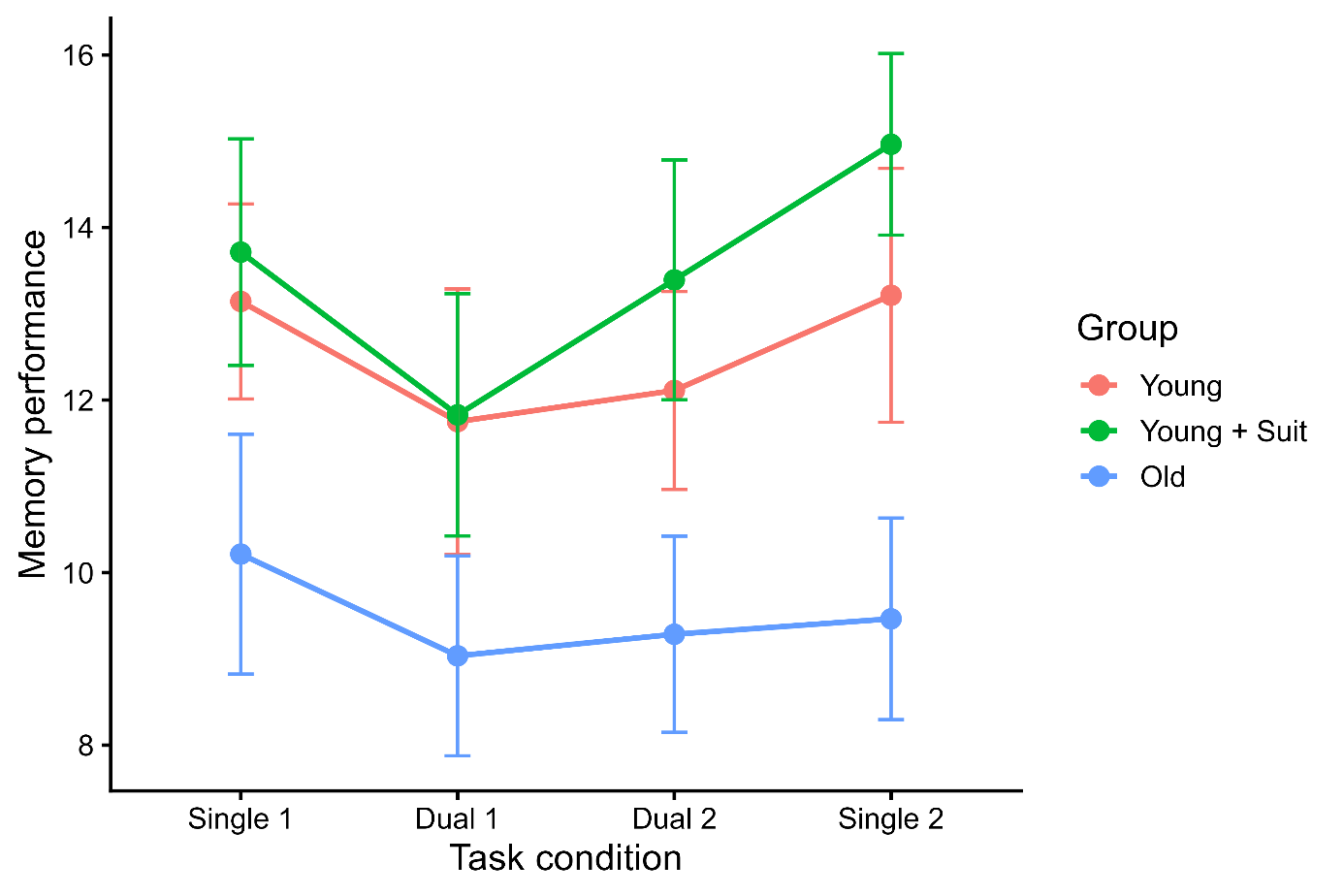
*

*Note.* Error bars depict SE of the mean.

**Figure S3.2**

*Gait Speed across the four task phases (Single 1, Dual1, Dual 2, Single 2) for the three groups*


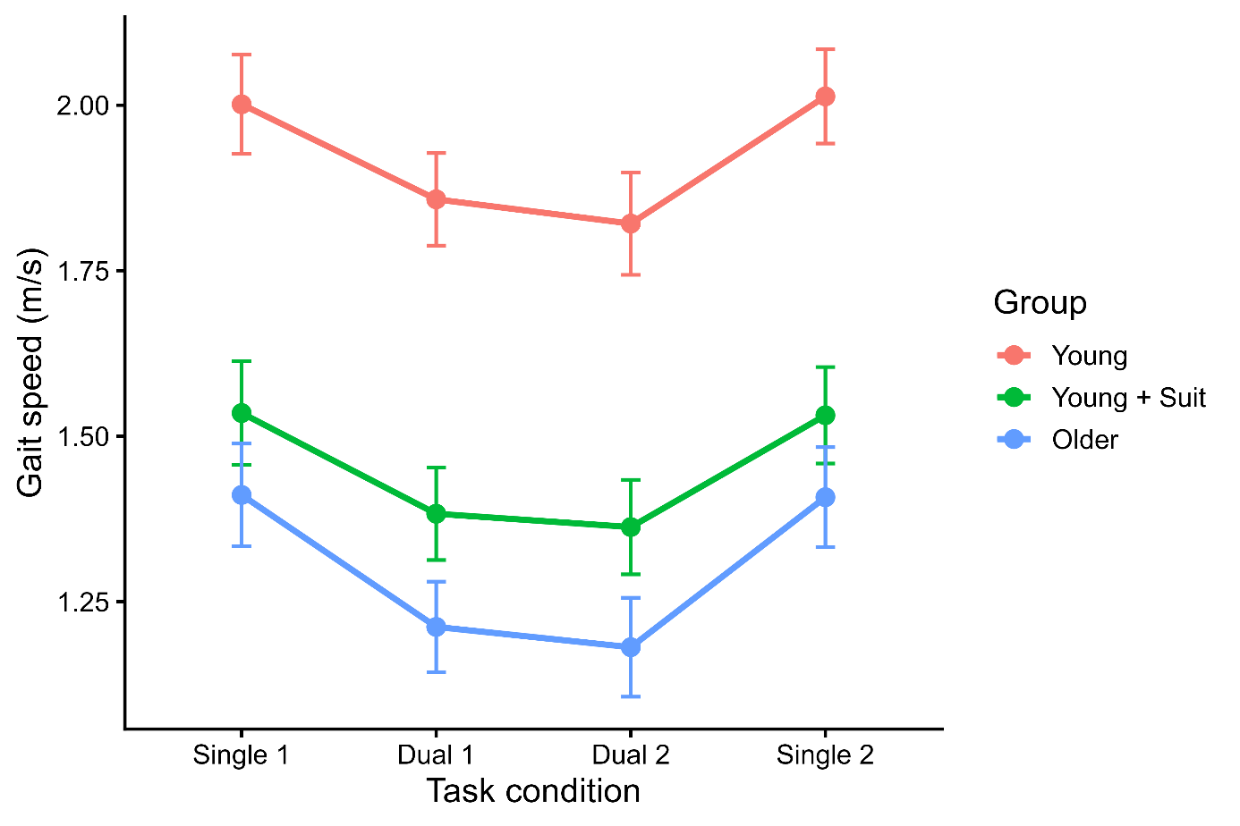


*Note.* Error bars depict SE of the mean.
